# Studying memory processes at different levels with simultaneous depth and surface EEG recordings

**DOI:** 10.3389/fnhum.2023.1154038

**Published:** 2023-04-04

**Authors:** Andrei Barborica, Ioana Mindruta, Víctor J. López-Madrona, F-Xavier Alario, Agnès Trébuchon, Cristian Donos, Irina Oane, Constantin Pistol, Felicia Mihai, Christian G. Bénar

**Affiliations:** ^1^Department of Physics, University of Bucharest, Bucharest, Romania; ^2^Epilepsy Monitoring Unit, Department of Neurology, Emergency University Hospital Bucharest, Bucharest, Romania; ^3^Department of Neurology, Medical Faculty, Carol Davila University of Medicine and Pharmacy Bucharest, Bucharest, Romania; ^4^Aix Marseille University, INSERM, INS, Institute of Neuroscience System, Marseille, France; ^5^Aix Marseille University, CNRS, LPC, Marseille, France; ^6^APHM, Timone Hospital, Epileptology and Cerebral Rhythmology, Marseille, France; ^7^APHM, Timone Hospital, Functional and Stereotactic Neurosurgery, Marseille, France

**Keywords:** EEG, stereo-EEG, simultaneous recordings, multivariate pattern analysis, recognition memory

## Abstract

Investigating cognitive brain functions using non-invasive electrophysiology can be challenging due to the particularities of the task-related EEG activity, the depth of the activated brain areas, and the extent of the networks involved. Stereoelectroencephalographic (SEEG) investigations in patients with drug-resistant epilepsy offer an extraordinary opportunity to validate information derived from non-invasive recordings at macro-scales. The SEEG approach can provide brain activity with high spatial specificity during tasks that target specific cognitive processes (e.g., memory). Full validation is possible only when performing simultaneous scalp SEEG recordings, which allows recording signals in the exact same brain state. This is the approach we have taken in 12 subjects performing a visual memory task that requires the recognition of previously viewed objects. The intracranial signals on 965 contact pairs have been compared to 391 simultaneously recorded scalp signals at a regional and whole-brain level, using multivariate pattern analysis. The results show that the task conditions are best captured by intracranial sensors, despite the limited spatial coverage of SEEG electrodes, compared to the whole-brain non-invasive recordings. Applying beamformer source reconstruction or independent component analysis does not result in an improvement of the multivariate task decoding performance using surface sensor data. By analyzing a joint scalp and SEEG dataset, we investigated whether the two types of signals carry complementary information that might improve the machine-learning classifier performance. This joint analysis revealed that the results are driven by the modality exhibiting best individual performance, namely SEEG.

## 1. Introduction

Electroencephalography (EEG) is routinely used to understand cognitive processes (Kappenman and Luck, 2011). The ability of these non-invasive recordings to capture cognitive processes accurately and entirely is the subject of ongoing investigations. A primary challenge is the well-known ill-posed problem of source reconstruction (Grech et al., 2008). Knowing the actual sources and their time course in detail would provide invaluable information to disentangle brain activities. Clinical uses of EEG face a similar challenge, e.g., concerning the surface visibility of epileptiform activity, either ictal or inter-ictal. The challenge has been addressed through the simultaneous recording of intracranial and surface, both with EEG (Tao et al., 2005; Ray et al., 2007; Koessler et al., 2015; Antony et al., 2019; Barborica et al., 2021) and MEG (Pizzo et al., 2019). An asset of the clinical context is that many forms of epileptiform activity, sometimes paroxysmal, involve relatively large patches of cortical tissue that present synchronized activity, evoking potentials on the scalp having reasonable signal-to-noise ratio (SNR). By contrast, cognitive processes evoke more subtle activities and variations, involving deep brain structures, and high-frequency activity. These factors may cumulatively contribute to poor scalp visibility of the corresponding EEG activity.

Recognition memory (Yonelinas, 2002) provides an ideal test case to explore how neural activities evoked by cognitive tasks are captured at the scalp by EEG. Recognition memory is a complex cognitive function generally broken down into encoding, storage, and retrieval processes (Mandler, 1980; Besson et al., 2012). These are known to involve lateral and deep structures such as the hippocampus (Rutishauser et al., 2006; Merkow et al., 2015). Recognition memory has been extensively studied with EEG, using recordings made either on the scalp (Ratcliff et al., 2016) or in the brain (Merkow et al., 2015), but not simultaneously. Here, we assess to what extent the postulated processes are visible on scalp EEG by validating the source localization results with the simultaneous scalp intracranial recordings. The data are collected from patients undergoing stereoelectroencephalographic (SEEG) pre-surgical evaluation for drug-resistant epilepsy; they performed a standard task requiring them to encode and later recognize pictures of objects (Besson et al., 2012; Despouy et al., 2020).

In animal studies, memory processes have been widely studied using electrophysiological recordings. The reduced size of the brain limits the number of electrodes that can be implanted in the behaving animal. Therefore, most studies include recordings from only one or two regions, mainly in the hippocampal formation (Mizuseki et al., 2009; López-Madrona et al., 2020). However, their spatial resolution is much higher than human SEEG, with a single electrode composed of up to seven shanks in the array (Csicsvari et al., 2003) or containing hundreds of recording channels (Steinmetz et al., 2021).

For our data collected simultaneously at different scales (mesoscale – SEEG, macroscale – scalp) in human subjects, providing wide (SEEG) or whole-brain (surface EEG) spatial coverage, we have performed a high-sensitivity multivariate pattern analysis (MVPA) (Haxby et al., 2001; Grootswagers et al., 2017), not only on individual sets of signals of different modalities (intracranial, scalp, or reconstructions) but also on combined sets having higher dimensionality, to evidence possible synergies between signals recorded at different scales.

## 2. Methods

### 2.1. Subjects

We selected 12 patients diagnosed with focal drug-resistant epilepsy that underwent long-term simultaneous EEG and SEEG recordings in the Emergency University Hospital Bucharest between 2020 and 2022 (Table 1). Patients were considered surgical candidates and underwent pre-surgical non-invasive evaluation using extended patient history, video-electroencephalography, brain structural and functional imaging (inter-ictal FDG-PET CT), and neuropsychological profile. Consequently, in these patients, invasive recordings were considered necessary to delineate the epileptogenic zone and to map the functional cortex for tailoring the surgical resection (Munari et al., 1994; Kahane et al., 2003; Jayakar et al., 2016; Isnard et al., 2018). The details regarding the patients' gender, age, type of epilepsy, and lateralization are provided in Table 1. In addition, as part of this research protocol, scalp electrodes were attached, allowing for simultaneous surface and intracranial long-term recordings. This study included only patients with unmodified anatomy, no previous major resection, and no major cognitive deficit.

**Table 1:** Patients included in this study.

**Patient**	**ID**	**Age**	**Epilepsy**	**Laterali zation**	**Language organization**	**SEEG electrodes**	**SEEG contacts**	**Scalp electrodes**	**SEEG electrode location**
1	89	37	Insular	R	Left typical	14	172	30	Left, posterior
2	90	17	Insular-opercular	L	Left typical	9	86	30	Left, central
3	92	27	Insular	L	Left typical	10	145	30	Left, posterior
4	96	26	Temporal	R	Left typical	11	152	38	Right, anterior
5	97	26	Rolandic Operculum	L	Atypical bilateral	10	135	35	Left, central
6	98	39	Temporal	R	Left typical	9	129	38	Right, posterior
7	99	24	Insular	L	Left typical	13	189	38	Left, anterior
8	101	31	Temporal	B	Left typical	14	187	40	Bilateral, central
9	102	31	Temporo-insular	B	Left typical	16	229	40	Bilateral, posterior
10	104	20	Insular	L	Left typical	10	124	40	Left, central
11	105	26	Frontal	R	Left typical	12	161	37	Right, anterior
12	107	26	Frontal	L	Left typical	8	176	40	Left, anterior

The study has been performed under Bucharest University ethical committee approval CEC 23/20.04.2019. All patients, or their legal guardian/next of kin, signed a written informed consent, in accordance with the Declaration of Helsinki, for the simultaneous recordings and data sharing procedures.

### 2.2. Experimental paradigm

We used the same experimental visual memory paradigm as in López-Madrona et al. (2022). In summary, we used 168 images from the database of Duñabeitia et al. (2018) that were organized in blocks of 12 or 24 images, presented on a computer screen. There were two block types: encoding (“ENC”), where a set of 12 images were presented to the patient, followed by a recognition block where the same 12 familiar images (“OLD”) were randomly interleaved with other 12 novel images (“NEW”). The patient was required to indicate by pressing two buttons on the keyboard, using two fingers of the right hand, whether the images were familiar or not, within 1500 ms. A distracting video of 1 min was presented in between encoding and recognition blocks. The sequence of 36 image presentations was repeated seven times using different images from the 168-image set and pseudo-random distribution of the OLD and NEW items, with the constraint that there were never more than three “old” or “new” items in a row. Stimuli presentation and response logging were controlled by the software E-Prime 3.0 (Psychology Software Tools, Pittsburgh, PA).

### 2.3. Simultaneous scalp and intracranial recordings

Stereoelectroencephalographic exploration was performed using depth electrodes (Dixi Medical, Chaudefontaine, France) with 8 to 18 contacts per electrode, 2 mm contact length, 3.5 mm center-to-center contact spacing, and 0.8 mm diameter. Multiple electrodes (9 – 16) were placed following a patient-specific hypothesis regarding the localization of the seizure onset zone and the pathways of ictal spread (Kahane et al., 2003; Jayakar et al., 2016), allowing for up to 229 contacts to be available in each patient. Electrodes were placed intracranially using the microTargeting™ Multi-Oblique Epilepsy STarFix Platform (FHC, Bowdoin, ME USA) (Dewan et al., 2018; Yu et al., 2018; Pistol et al., 2021) or the Leksell stereotactic frame (Elekta AB, Stockholm, Sweden). To determine the exact location of each electrode and contact, the post-implantation CT scan was loaded into surgical planning software (Waypoint Planner, FHC, Bowdoin, ME USA), co-registered with the pre-implantation MRI, and adjustments to the initially planned trajectories were made to match the postop location of the electrodes. Manual labeling of the SEEG contacts has been performed using the abbreviations listed in Supplementary Table S1.

In view of the group analysis, the pre-surgical MRI of each patient was also used for running an analysis pipeline implemented in FreeSurfer (Fischl, 2012) that allowed us to obtain the patient's cortical surface reconstruction, used for visualization purposes, but also—more importantly—, for performing a non-rigid registration of the patient's MRI to the “cvs_avg35_inMNI152” FreeSurfer template (Postelnicu et al., 2009), providing us with the coordinates of each intracranial contact in a common MNI space.

One up to three days after the SEEG implantation, between 30 and 40 scalp electrodes were placed according to the 10-20 system. A few electrodes were repositioned on adjacent 10-10 grid location, due to interference with the SEEG electrodes, and up to 10 electrodes could not be placed at all. The exact number of scalp electrodes in each patient is provided in Table 1.

**Figure 1:**
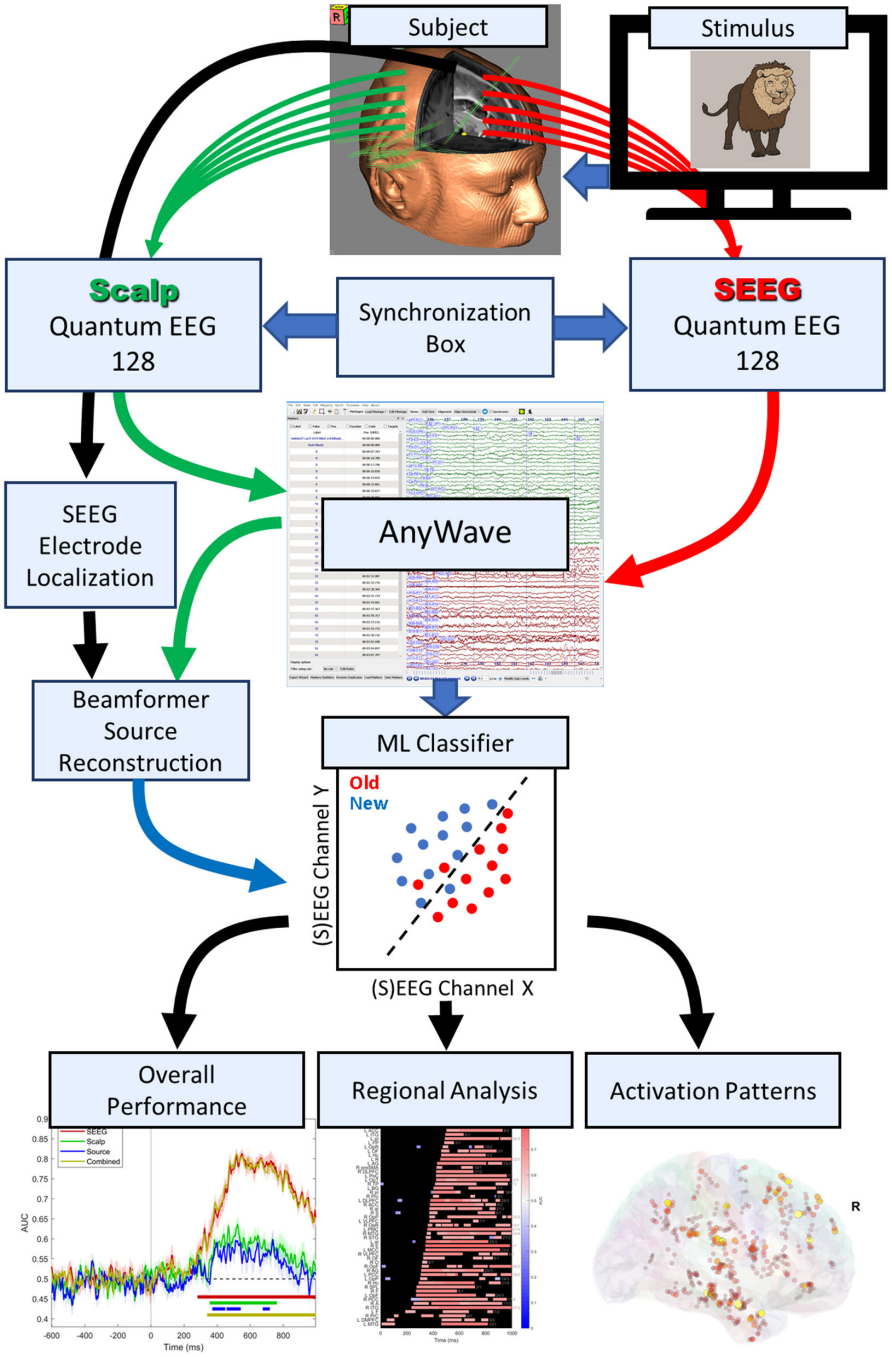
Signal collection and analysis workflow.

Signals were collected using a setup as described by Barborica et al. (2021). In summary, two identical Natus Quantum 128-channel amplifiers (Natus Neuro, Middleton, WI) were used, one for each modality (scalp/intracranial) and having separate signal references. The reference for the SEEG recordings was chosen on one contact located in white matter exhibiting minimal activity, whereas the reference for the scalp system was Fpz. Raw data were acquired at a sample rate of 4096 Hz. The hardware was synchronized using digital triggers to both systems and a 50 Hz sine reference signal, recorded simultaneously using DC inputs of the two systems. Patients 9–12 were recorded with a single Quantum 256-channel amplifier that did not require external synchronization hardware. The data were combined and saved in a single file in AnyWave ADES format (Colombet et al., 2015), containing both types of signals. The analysis workflow is shown in Figure 1.

The synchronization between stimuli presentation and (S)EEG recordings has been performed using a photodiode part of the Chronos response box (Psychology Software Tools, Pittsburgh, PA) attached to a corner of the screen where trial start synchronization flashes were presented. The response time and correctness were merged into the AnyWave event file by reading the E-Prime log files using MATLAB (MathWorks, Natick, MA) custom scripts.

Only intracranial sensors located outside the seizure onset zone and gray matter were included in the analysis. Additional artifacted trial removal, as well as bad channel removal, was performed manually by visually inspecting the recordings.

### 2.4. ERP processing

Signals were loaded into EEGLab (Delorme and Makeig, 2004) software, resampled at 256 Hz, and filtered in the 0.5–45 Hz interval. Scalp EEG was re-referenced to the common average, and artifacts were removed using independent component analysis (ICA). Only correct trials have been retained for further analysis.

### 2.5. Source localization

To test the inverse solution of scalp EEG for finding brain areas that are involved in task decoding, we have calculated source signals at the location of the intracranial electrodes. To achieve that, we have performed a beamformer analysis on the standard FreeSurfer's *fsaverage* template, brain electrical model, and 10–20 electrode positions available in MNE-Python (Gramfort et al., 2014). The beamformer spatial filters calculated using linearly constrained minimum variance (LCMV) (Van Veen et al., 1997) were used to calculate source time courses on a 5-mm grid covering the brain. The source time course on the grid point nearest to the midpoint between a pair of SEEG contacts that were part of a bipolar-recorded signal was considered to approximate the source signal at each intracranial site. We, therefore, obtained a set of signals with the same dimensionality as the SEEG, which we analyzed using the common MVPA pipeline. Calculating the source signals at the locations near intracranial recording sites allowed the SEEG recordings to be used as ground truth for source reconstructions (Mikulan et al., 2020) and provided the ability to compare task-related activations at the same locations for both types of signals.

### 2.6. Independent component analysis

To test whether a method that is known to separate temporally correlated neuronal sources can enhance MVPA decoding results, we have performed an independent component analysis (ICA) of scalp signals using second-order blind identification (SOBI) blind source separation (Belouchrani et al., 1993, 1997; Tang et al., 2005), using EEGLab software (Delorme and Makeig, 2004).

### 2.7. Multivariate pattern analysis

For multivariate pattern analysis, we have generally followed the workflow described in Grootswagers et al. (2017). The processing has been performed using the MNE-Python toolbox (Gramfort et al., 2013, 2014) and custom Python and MATLAB (MathWorks, Natick, MA) scripts. A logistic regression linear classifier was trained to discriminate between responses for the OLD and NEW conditions using the L-BFGS-B – (Large-scale Bound-constrained Optimization) solver. The model was fitted to the standardized data, and its performance was scored using the receiver operating characteristic (ROC) area under the curve (AUC). The scores were evaluated using 20-fold cross-validation, and time intervals where they were statistically different from chance were evaluated using a one-sample permutation cluster test applied to the set of scores calculated for each fold (Maris and Oostenveld, 2007).

The processing pipeline was applied to SEEG bipolar signals, to the EEG signals, to the entire set of independent components of scalp EEG, or to the scalp source signals at the SEEG sensor location obtained using a beamformer. Specific to our study, the simultaneous collection of the scalp and SEEG data allowed the pooling of the signals for the two modalities to investigate whether combined data provide a classifier performance significantly different from analyzing individual sets.

We have calculated the contribution of signals at each intracranial sensor location (recorded or reconstructed) to the recognition process by calculating the activation patterns associated with fitting the data with a linear model (Haufe et al., 2014) using the MNE-Python toolbox, which in turn resorts extensively to scikit-learn Python toolbox (Pedregosa et al., 2011; Abraham et al., 2014). In contrast to the classifier weights associated with each sensor, which do not have a direct interpretation, the reconstructed activation patterns are interpretable as neural sources encoding the studied processes that can be projected onto the sensors (Haufe et al., 2014; Fahrenfort et al., 2018).

To assess the contribution of various brain structures to decoding task conditions, we repeated the MVPA analysis on a subset of signals recorded or reconstructed within the same brain area or structure (Despouy et al., 2020), according to the labeling we have described earlier in this section. We will further refer to this analysis restricted to a region of interest (ROI) as “regional analysis” (Ebrahiminia et al., 2022). In contrast to activation patterns (Haufe et al., 2014), which have no statistical significance associated with their time courses, regional analysis allows inferring, in a probabilistic way, the time intervals where decoding performance is different from chance, evidencing the sequential/hierarchical processing of stimulus novelty within the brain.

## 3. Results

A total of 136 intracranial electrodes having 1,885 contacts were implanted in 12 patients. Additional 436 surface electrodes were attached to the scalp. After data curation and application of inclusion criteria, signals recorded at 965 intracranial sites and 391 scalp locations were further included in the analysis. The subjects correctly identified stimulus novelty in 89.53% of the trials. The MVPA analysis was applied to 1,729 correct recognition trials (OLD: 822, NEW: 907) having a mean ± SD response time of 719.1 ± 162.4 ms (OLD) and 765.0 ± 191.2 ms (NEW).

### 3.1. Responses on the single scalp and SEEG electrodes

**Figure 2:**
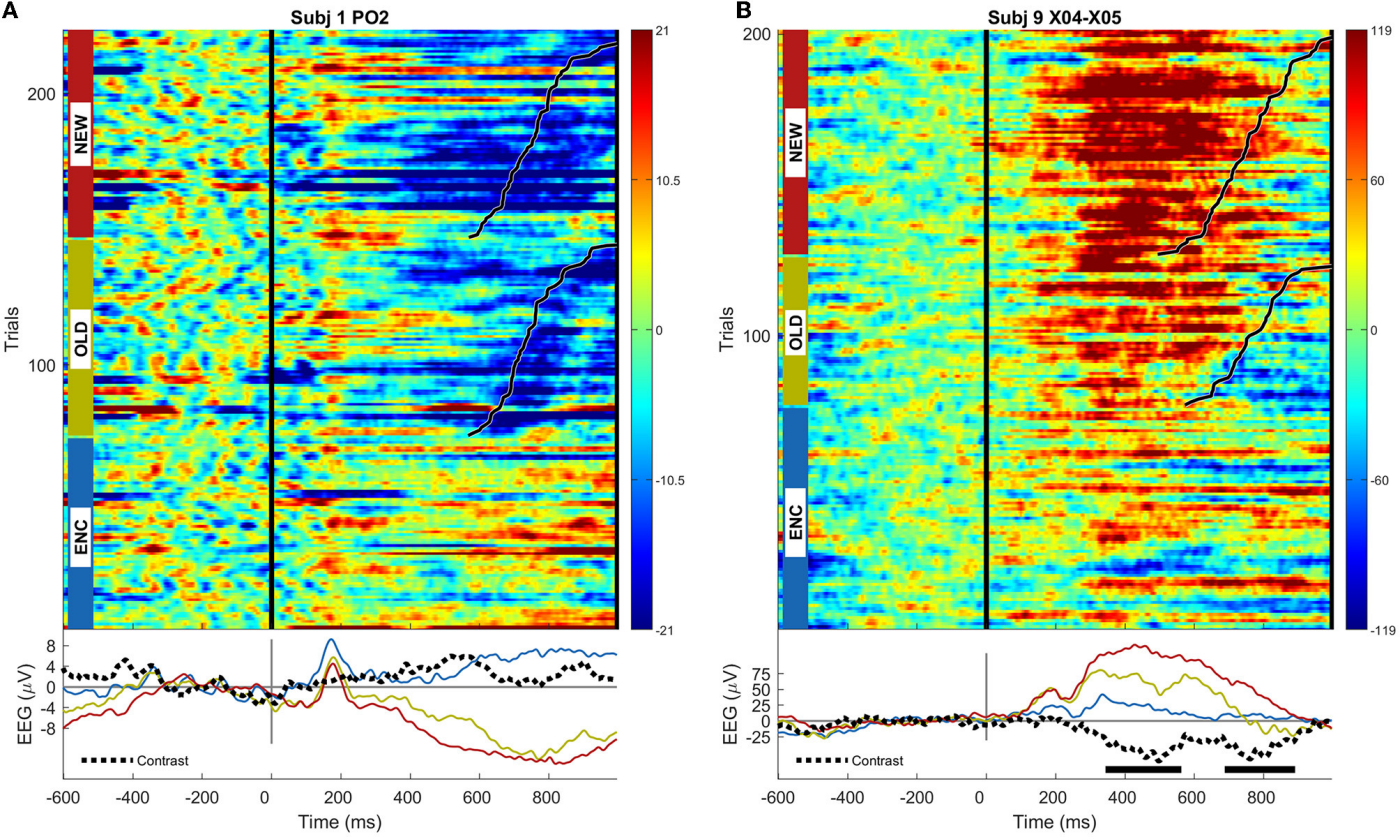
**(A)** ERP image for the scalp sensor PO2 in subject 1, exhibiting the highest multivariate activation pattern; trials are grouped by condition and sorted by the response time, marked using black lines; average ERPs for each condition, as well as the contrast between OLD and NEW conditions, are shown; the statistical significance of the univariate (permutation cluster test) difference, if present, between OLD and NEW conditions at a significance level *p* < 0.05 is shown using thick horizontal lines; **(B)** same as **(A)**, but for the intracranial sensor X04-X05 located in right anterior insula in subject 9.

The ERPs for the scalp sensor and SEEG sensor having the highest magnitude multivariate activation patterns among all scalp and *m* = 965 SEEG signals recorded in all *n* = 12 patients are shown in Figure 2.

While a typical high-amplitude ERP presents prominent peaks either following the stimulus presentation (~200 ms) or around response time, depending on sensor location, these examples rather capture situations where the novelty of the stimulus is best captured, between 400 ms and 600 ms and around the response time (~800 ms).

### 3.2. Single-subject multivariate analysis

**Figure 3:**
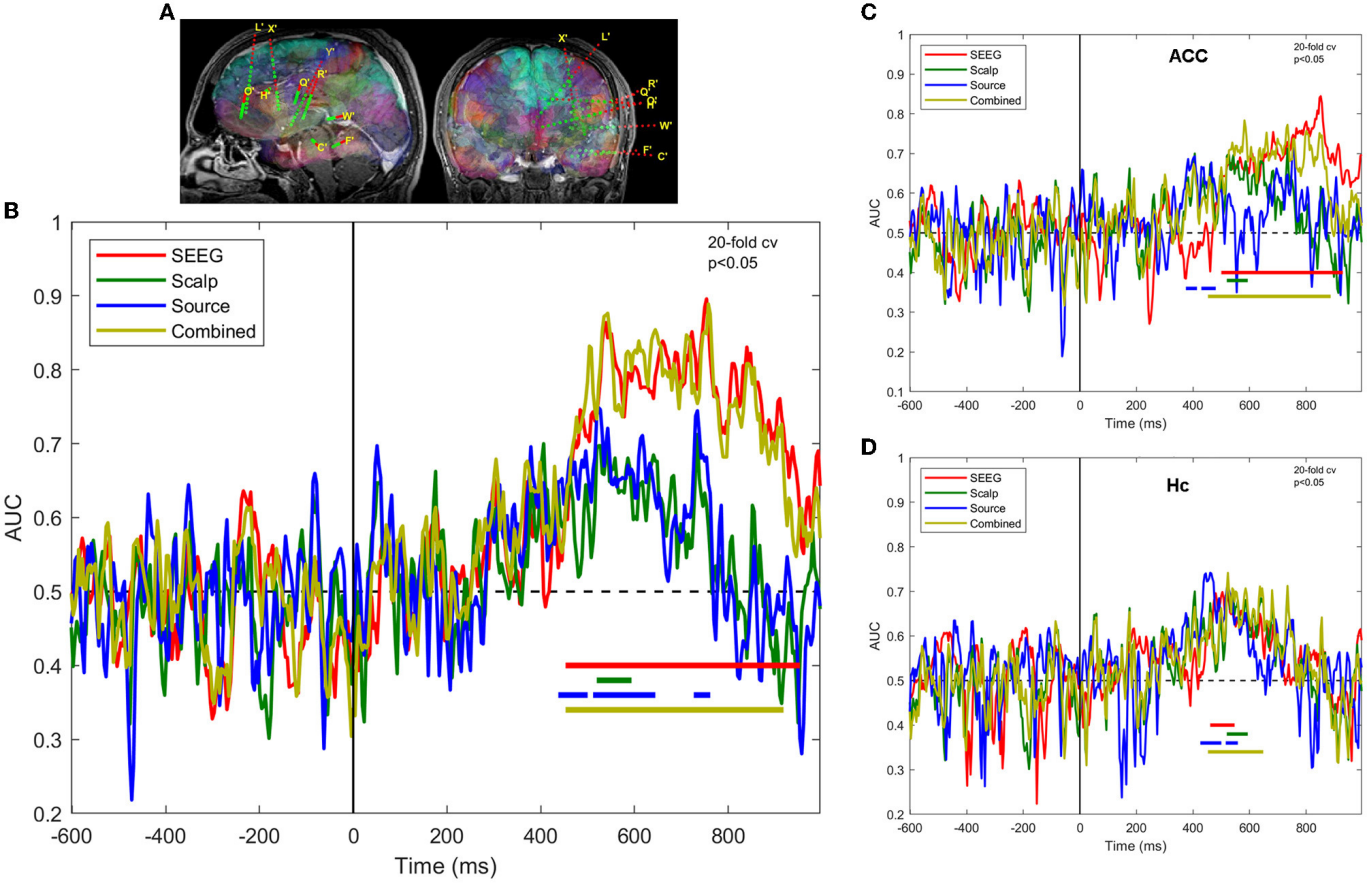
Task decoding performance is expressed as the area under curve of the receiver operating characteristic of the classifier for SEEG, scalp, and source signals in patient 3. **(A)** SEEG electrode locations in the left hemisphere; **(B)** ROC-AUC for sensors of different types, as well as for combined scalp and SEEG; **(C)** same as **(B)**, but for the contacts located in the anterior cingulate cortex; **(D)** same as **(B)**, but for contacts located in the hippocampus.

The results of the MVPA analysis of responses at the SEEG, scalp, source level, and combined scalp SEEG in patient 3 are shown in Figure 3. The classifier performance for the SEEG signals is consistently above-chance through the interval ~450 ms through ~900 ms (permutation cluster test, *p* < 0.05). By contrast, the scalp signals provide a statistically significant classification performance only during the memory retrieval and stimulus recognition processes between ~500 ms and ~600 ms. Computing source signals at SEEG sensor locations provide classification results that are similar in magnitude to the scalp sensor signals, with eventually better results in terms of the extent of the clusters reflecting the scores significantly different from chance (*p* < 0.05).

A regional MVPA analysis presented in Figures 3C, D highlights the regions that contribute most to the overall decoding performance, namely the anterior cingulate cortex and hippocampus. The ACC, as sampled by SEEG, exhibits sustained better-than-chance scores in the late interval ~500 ms through ~900 ms, whereas Hc presents early (~500 ms), but limited duration (~100 ms) activations. The scalp, source, and combined signals provide similar results in Hc, but rather different ones in ACC.

### 3.3. Group analysis

**Figure 4:**
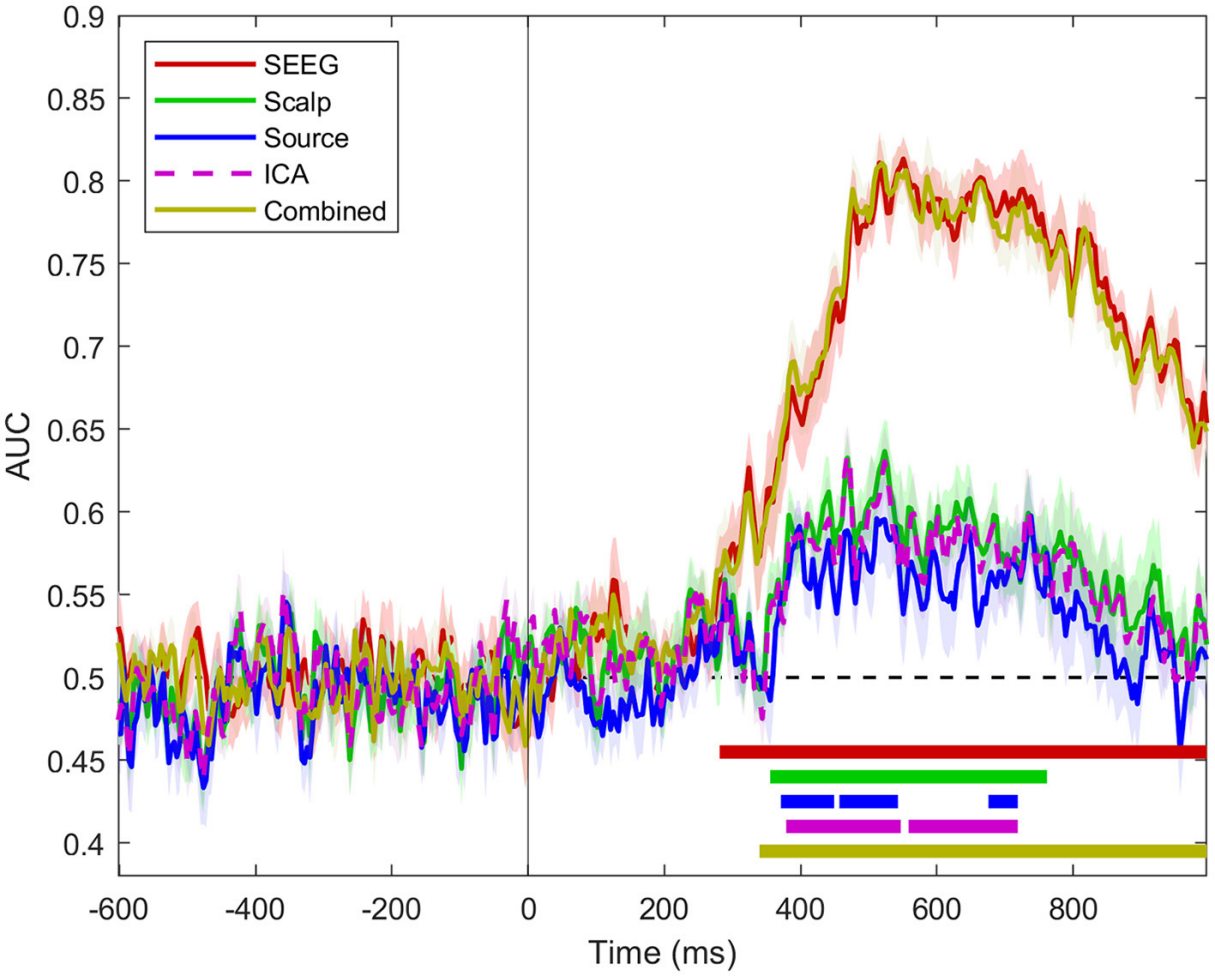
Classifier performance for SEEG, scalp, source, ICA, and combined scalp SEEG signals for all *n* = 12 subjects. The dashed areas show standard error intervals for the set of classifier scores for all patients. The horizontal bars indicate the intervals where the scores are statistically different from chance (one-sample permutation cluster test, *p* < 0.05).

At the population level (*n* = 12 subjects), the classifier performance based on intracranial signals was much higher than the one based on scalp or source signals, as shown in Figure 4.

The use of source signals calculated at SEEG sensor locations provides slightly lower classifier performance than the one based on signals from which it was derived, i.e., scalp signal (Figure 4). The MVPA analysis applied to the independent components of the scalp signal provides results that are virtually identical to the scalp ones. Combined scalp and SEEG scores follow closely the time course of the SEEG scores.

**Figure 5:**
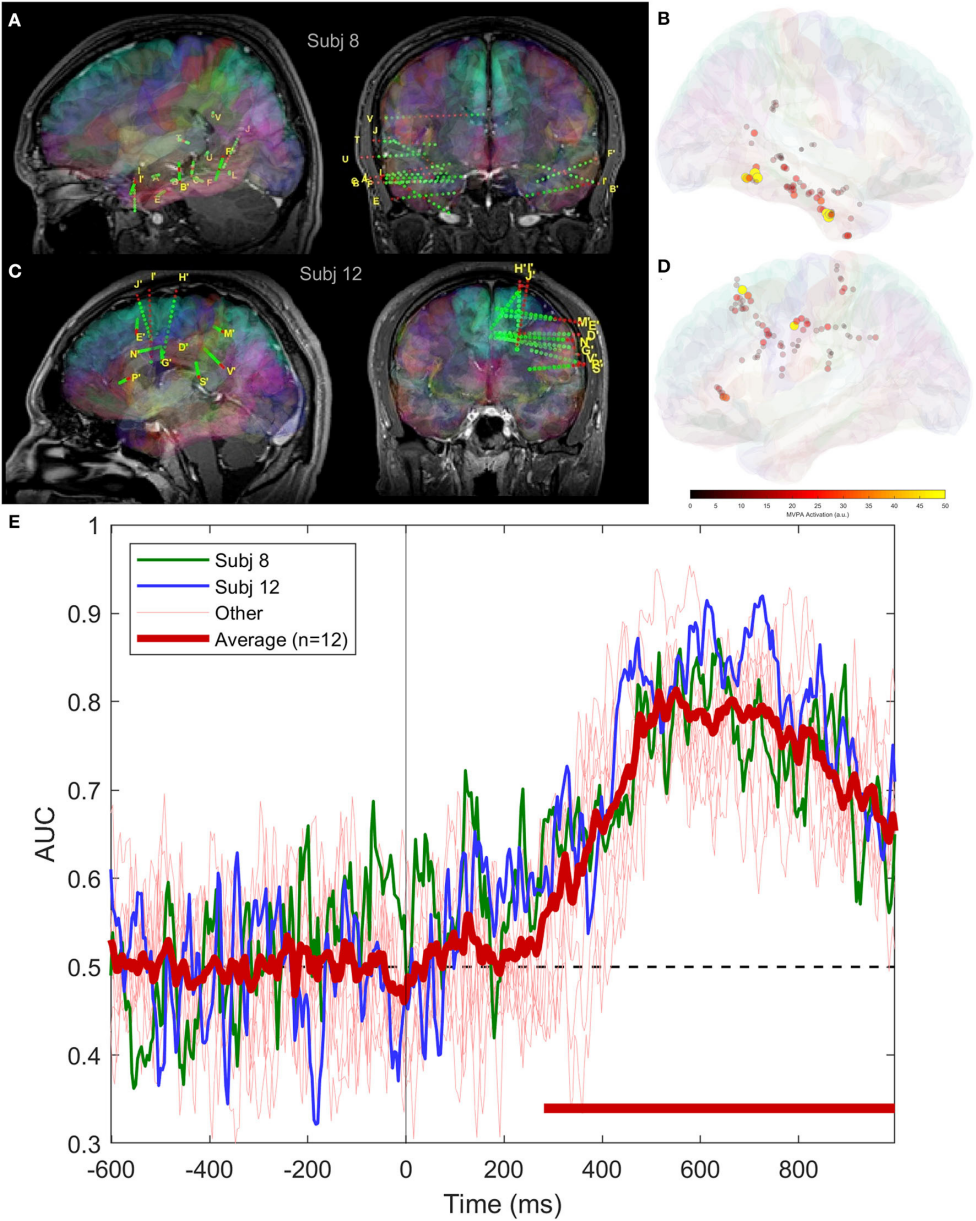
Classifier performance using intracranial signals for two patients having SEEG implantation covering different areas of the brain; **(A)** bilateral implantation in subject 8, covering temporal lobe, including mesial structures; **(B)** mean magnitude of activation patterns in subject 8 across the entire trial duration; **(C)** electrode locations in subject 12, frontal, parietal and cingulate areas; **(D)** same as **(B)**, but for subject 12; and **(E)** average and individual classifier scores.

The time course of classification performance using SEEG signals is consistent across subjects, as shown in Figure 5, where we have plotted the scores for all subjects as well as the grand average. This is somehow unexpected, as the areas implanted with depth electrodes can be quite different. In Figures 5A, C, we show two implantation schemes providing similar scores, which are highlighted in Figure 5E with green and blue colors.

**Figure 6:**
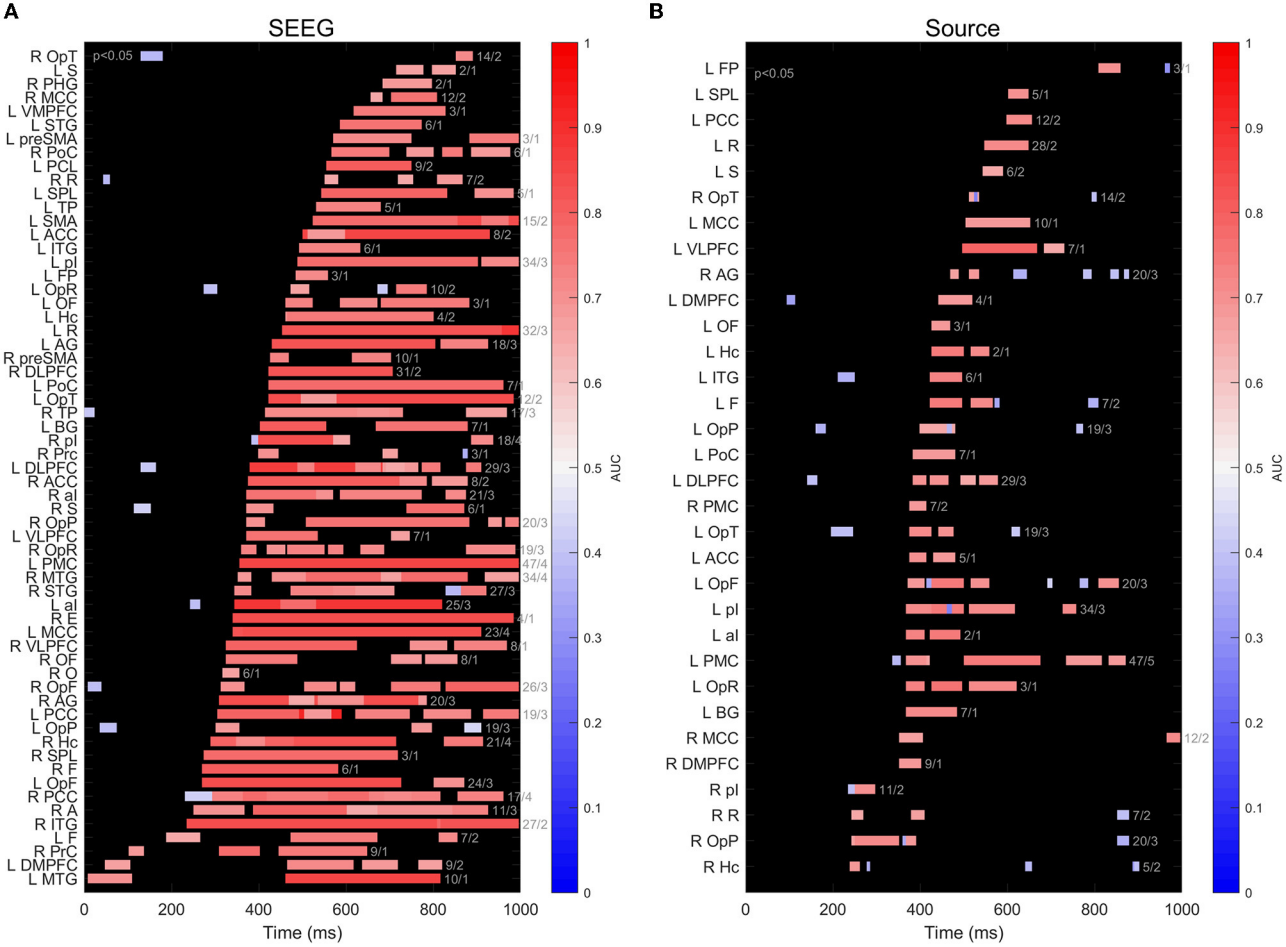
**(A)** Timeline of decoding performance significantly different from chance (*p* < 0.05) for signals recorded on subsets of intracranial contacts implanted in different brain structures. The color of the bars indicates the maximum value of the AUC score within a cluster. The numbers at the right of each bar indicate the number of sites and number of patients for clusters in each ROI; **(B)** same as **(A)**, but for scalp sources calculated at the location of intracranial contacts using beamformer.

When we perform a regional analysis of the performance in decoding task conditions, we see that the findings at the level of all *m* = 965 sites in *n* = 12 subjects, shown in Figure 4, are confirmed at a regional scale (Figure 6), with scores significantly different from chance associated with SEEG signals being higher and more sustained over time, compared to source signals reconstructed at the same locations. Among the areas exhibiting the highest and earliest SEEG scores, we can count F, ITG, and Hc, as well as the insular-opercular complex. One has to keep in mind that all these findings are strongly influenced by the coverage of each ROI with SEEG electrodes.

**Figure 7:**
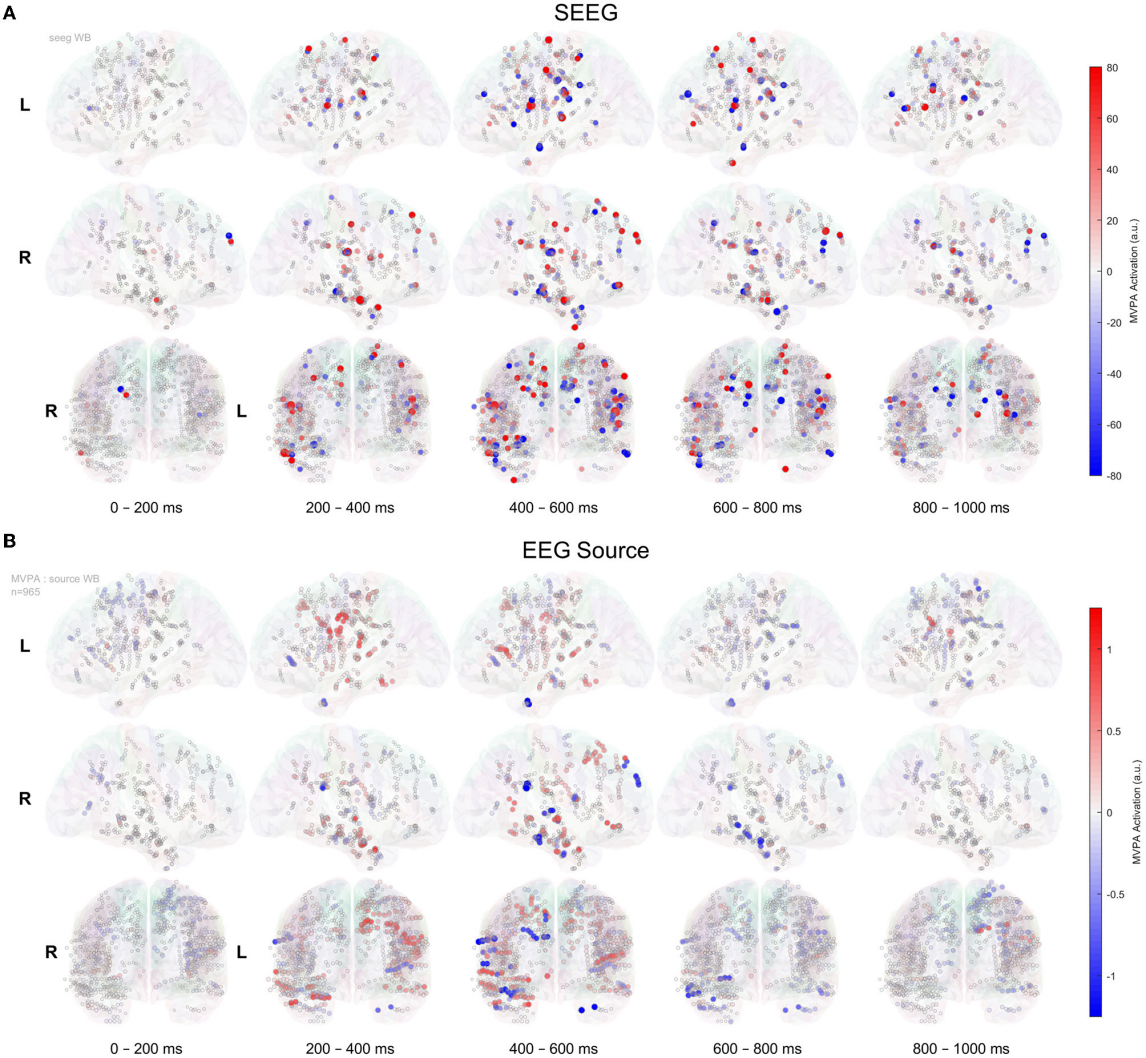
MVPA timeline of activation patterns, as introduced by Haufe et al. (2014), for SEEG signals **(A)** and in EEG source space **(B)** for all 965 contacts implanted in 12 patients, shown on the glass brain. The mean of activation patterns' values within 200 ms bins is represented.

The 3D representation of multivariate activation patterns (Haufe et al., 2014) of SEEG and source-space data is shown in Figure 7. One has to keep in mind that these activation patterns do not reflect the magnitude of the ERPs, but rather represent a virtual signal corresponding to how well a site encodes the stimulus novelty, in our case (Wardle et al., 2016; Grootswagers et al., 2017). A wide-area brain activation (Figure 7A) over the course of the recognition process is visible for the intracranial signals; whereas at a comparable amplitude scale, the source data show much less activations. The activation patterns of various brain areas are sequential, following a posterior-to-anterior flow, as illustrated in Figure 7 and in the Supplementary Data Movie. The activations associated with EEG source signals show a roughly similar spatiotemporal pattern. At a closer visual inspection of Figure 7, we can find evidence of known leakage-related effects (Schoffelen and Gross, 2009), as multiple contacts in several electrodes exhibit similar activation values.

In SEEG recordings (Figure 7A), we can divide the activation into four clinically relevant time intervals. The significant activation starts at ~20 0ms and between 200 and 400 ms, we can observe the recognition process that activates the network of structures that mainly involves the temporal-basal and hippocampus on the right side. Then, between 400 and 600 ms, we can see the activations related to the decision-making process that significantly involves bilaterally the perisylvian, prefrontal, and mesial temporal lobe structures. The sensorimotor activation overlaps the 400–600 ms and continues in the next interval of 600–800 ms and represents the response phase of the task. The last time interval (600–800 ms) highlights the activation of the prefrontal cortex possibly related to self-evaluation or memory storage. The EEG source (Figure 7B) displays a similar timeline of the activation pattern in the 200–600 ms. However, the late phase between 600 and 800 ms is not informative.

## 4. Discussion

While other studies comparing intracranial to scalp data used a sequential recording of the two modalities (Ebrahiminia et al., 2022) or even different sets of participants (Haufe et al., 2018), we have simultaneously acquired data in the two modalities, allowing us to validate the results of the EEG source reconstruction using SEEG recordings in decoding task conditions, as well as investigate the possible synergy between invasive and non-invasive recording in decoding stimulus novelty.

Our results show that our task requiring the subjects to categorize visual stimuli based on novelty, involving memory encoding and retrieval, activates large areas of the brain. This finding is supported by the widespread activation visible in Figure 7, as well as by the fact that SEEG implantations at totally different locations result in decoding performances over time that are close to each other and to the group average (Figure 5).

The decoding performance of the ML classifier is maximal when using intracranial signals, although the SEEG implantation has limited spatial coverage of the brain, compared with the scalp EEG which is supposed to provide full-brain coverage, as visible in Figures 3, 4. The relatively poor decoding performance of the classifier that uses scalp signals can be attributed, in our opinion, to the significantly lower signal-to-noise ratio (SNR) of the scalp EEG compared to SEEG. It is also possible that scalp EEG provides poor visibility of activity in deep structures of the brain, whereas SEEG samples with undegraded SNR all implanted locations, regardless of depth. A previous study by Ebrahiminia et al. (2022) performing sequential scalp and electrocorticographic (ECoG) recordings has shown that scalp EEG provides slightly better classification performance of passively viewing visual stimuli of different categories (Liu et al., 2009). Without counting the differences in the tasks, one reason for this discrepancy may relate, once again, to the fact that ECoG does not record activity in deep brain structures, therefore both modalities provide information from the outer cortex, with scalp EEG providing slightly better spatial coverage. Another factor that may favor EEG in other studies is that in our simultaneous protocol, the EEG electrodes were glued to the scalp 1 day or more before running the memory task (part of a wider set of investigations), resulting in a degradation of the quality of the contact within this interval, uncorrectable due to the requirement of maintaining sterility at the scalp level. Furthermore, due to spatial constraints related to pre-existing SEEG electrode anchors, the coverage with scalp electrodes was non-uniform.

Interestingly, using SEEG electrodes, the classifiers were always able to decode the task conditions using task-evoked intracranial EEG recorded 300 to 1000 ms post-stimuli presentation. This was true not only at the group, but also at the individual subject level, even when the spatial sampling of the SEEG electrodes was completely different (Figure 5). Recent studies have shown the “traveling wave” behavior of brain activity (Lubenov and Siapas, 2009; Muller et al., 2014; Liang et al., 2021; Bhattacharya et al., 2022), and it is possible that we have observed such effects in our analysis. Under the assumption that the task-evoked intracranial EEG activity is recorded on a critical number of electrodes, sufficient for the classifier to learn the propagation patterns of the traveling wave, we may decode the task conditions from various brain regions, without a loss in decoding performance. Similar effects have been observed by groups that studied the representation and processing of emotion in the brain with machine learning methods, concluding that emotion representation is encoded as patterns of activations over widely distributed brain networks (Wager et al., 2015; Donos et al., 2022).

The process of reconstructing the EEG source signals using beamforming does not result in a significant improvement at the population level of the classifier's performance, yielding results comparable to signals on scalp sensors, as shown in Figure 4. There are exceptions to that general finding in some individual patients, as illustrated in Figure 3B, where the decoding performance of a classifier operating on source signals show earlier and longer statistically significant above-chance scores than sensor-based analysis, at a significance level of *p* < 0.05. However, such results have to be treated with caution, given the probabilistic nature of the statistical tests applied (Sassenhagen and Draschkow, 2019). The regional analysis of the classification performance shown in Figure 6 is in agreement with the overall results in Figure 4, where source signals result in more sparse and limited-duration significant scores than the intracranial signals.

The beamformer source reconstruction is based on linear matrix operations on the responses (Westner et al., 2022). A linear transformation based on a square non-singular matrix is equivalent to an affine transformation in the n-dimensional response space, which is the space in which the MVPA operates (Grootswagers et al., 2017).

Another approach that also uses linear matrix transformations for separating statistically independent components in a set of signals is the independent component analysis (ICA). We have tested whether applying ICA to the scalp EEG responses, results in a set of independent components that provide better decoding of the task conditions. The results, presented in Figure 4, show that classifier performance operating on the full set of independent components, without dimensionality reduction, is virtually identical to the one for the original signals on the scalp sensors.

These data-driven findings related to deriving virtual signals/components based on linear transformations suggest more general theoretical considerations: a non-singular affine transformation, equivalent to a series of elementary transformations such as rotation, scaling, and shear (Goldman, 1992), that do not change the relationships between points representing the set of *n* responses at a particular point in time; therefore, it is not expected to significantly affect the performance of an ML classifier operating on the transformed set of points. It should be noted that only linear transformations that do not perform dimensionality reduction (i.e., the transformation matrix is square), such as the ones we have considered, are equivalent to affine transformations and thus unlikely to affect MVPA classifier accuracy, based on geometrical considerations. By contrast, other approaches based on non-square linear transformation matrices, such as principal component analysis with dimensionality reduction, have been shown to have the potential for improving ML classifiers' accuracy (Grootswagers et al., 2017; Hatamimajoumerd et al., 2020).

In investigating whether scalp and intracranial signals contain complementary information that might contribute to a classifier performance, we did find that the modality providing the best performance (i.e., SEEG) is determining the combined performance (Figures 3, 4).

A limitation of the study is the partial and non-uniform spatial sampling of both scalp and intracranial sensors, due to objective reasons. The source reconstructions are based on the *fsaverage* template and associated volume conduction model, without taking into account the individual patient anatomy, which might lead to different results (Ramon et al., 2006; Céspedes-Villar et al., 2020). The scalp electrode locations used for calculating inverse solution were the standard 10–20 ones, aligned with the *fsaverage* head model, without taking into account the displacements related to the inaccuracy of the electrode positioning and the avoidance of the SEEG electrodes. Only one source-reconstruction-algorithm with fixed parametrization, among many different possible ones, has been considered (Grech et al., 2008; Mikulan et al., 2020). Another limitation is that our analysis pipeline is the most conservative one, being based on wide-band single-trial data. Creating “super-trials” or “pseudo-trials” by averaging several trials (Despouy et al., 2020; Ashton et al., 2022) might improve the SNR of EEG and correspondingly of the source reconstruction signals. Further measures for improving SNR can be possibly implemented (Grootswagers et al., 2017), alleviating some of the apparent limitations of non-invasive recordings.

## 5. Conclusion

Analysis of invasive EEG provides the highest amount of information related to stimulus novelty, compared with scalp recordings, despite the limited spatial sampling of the brain with depth electrodes. This may be related to the limited scalp visibility of the activity related to memory processes in deep brain structures, particularly if containing higher frequency components. The synergy between the two modalities—enabled by pooling data recorded simultaneously—is limited, with the SEEG sensors providing the best decoding performance driving the combined, overall, performance.

## Data availability statement

The datasets presented in this study can be found in online repositories. The URL of the repository is: http://epi.fizica.unibuc.ro/scalesoldnew/.

## Ethics statement

The studies involving human participants were reviewed and approved by the University of Bucharest Ethics Committee for Research, approval number CEC 23/20.04.2019. Written informed consent to participate in this study was provided by the participants' legal guardian/next of kin.

## Author contributions

AB: conceptualization, methodology, software, formal analysis, resources, data curation, writing, visualization, supervision, and funding acquisition. IM: methodology, investigation, writing, and supervision. VL-M and AT: methodology. F-XA: methodology and writing—review and editing. CD: methodology, software, formal analysis, and writing—initial draft. IO: investigation. CP: investigation, formal analysis, and data curation. FM: investigation and data curation. CB: conceptualization, methodology, writing—review and editing, and funding acquisition. All authors contributed to the article and approved the submitted version.

## References

[B1] Abraham A. Pedregosa F. Eickenberg M. Gervais P. Mueller A. Kossaifi J.. (2014). Machine learning for neuroimaging with scikit-learn. Front. Neuroinform. 8, 14. 10.3389/fninf.2014.00014 24600388 PMC3930868

[B2] Antony A. R. Abramovici S. Krafty R. T. Pan J. Richardson M. Bagic A.. (2019). Simultaneous scalp EEG improves seizure lateralization during unilateral intracranial EEG evaluation in temporal lobe epilepsy. Seizure 64, 8–15. 10.1016/j.seizure.2018.11.015 30502684

[B3] Ashton K. Zinszer B. D. Cichy R. M. Nelson C. A. Aslin R. N. Bayet L. (2022). Time-resolved multivariate pattern analysis of infant EEG data: A practical tutorial. Dev. Cogn. Neurosci. 54, 101094. 10.1016/j.dcn.2022.101094 35248819 PMC8897621

[B4] Barborica A. Mindruta I. Sheybani L. Spinelli L. Oane I. Pistol C.. (2021). Extracting seizure onset from surface EEG with independent component analysis: Insights from simultaneous scalp and intracerebral EEG. NeuroImage. Clin. 32, 102838. 10.1016/j.nicl.2021.102838 34624636 PMC8503578

[B5] Belouchrani A. Abed-Meraim K. Cardoso J. F. Moulines É. (1993). Second Order Blind Separation of Temporally Correlated Sources. Proc. Int. Conf. Digit. Signal Process., 346–351.

[B6] Belouchrani A. Abed-Meraim K. Cardoso J. F. Moulines É. (1997). A blind source separation technique using second-order statistics. IEEE Trans. Signal Process. 45, 434–444. 10.1109/78.554307

[B7] Besson G. Ceccaldi M. Didic M. Barbeau E. J. (2012). The speed of visual recognition memory. Vis. cogn. 20, 1131–1152. 10.1080/13506285.2012.724034

[B8] Bhattacharya S. Brincat S. L. Lundqvist M. Miller E. K. (2022). Traveling waves in the prefrontal cortex during working memory. PLOS Comput. Biol. 18, e1009827. 10.1371/journal.pcbi.1009827 35089915 PMC8827486

[B9] Céspedes-Villar Y. Martinez-Vargas J. D. Castellanos-Dominguez G. (2020). Influence of patient-specific head modeling on EEG source imaging. Comput. Math. Methods Med. 2020, 5076865. 10.1155/2020/5076865 32328152 PMC7157795

[B10] Colombet B. Woodman M. Badier J. M. Benar C. G. (2015). AnyWave: a cross-platform and modular software for visualizing and processing electrophysiological signals. J. Neurosci. Methods 242, 118–126. 10.1016/j.jneumeth.2015.01.017 25614386

[B11] Csicsvari J. Henze D. A. Jamieson B. Harris K. D. Sirota A. Barthó P.. (2003). Massively parallel recording of unit and local field potentials with silicon-based electrodes. J. Neurophysiol. 90, 1314–1323. 10.1152/jn.00116.2003 12904510

[B12] Delorme A. Makeig S. (2004). EEGLAB: An open source toolbox for analysis of single-trial EEG dynamics including independent component analysis. J. Neurosci. Methods 134, 9–21. 10.1016/j.jneumeth.2003.10.009 15102499

[B13] Despouy E. Curot J. Deudon M. Gardy L. Denuelle M. Sol J. C.. (2020). A fast visual recognition memory system in humans identified using intracerebral ERP. Cereb. Cortex 30, 2961–2971. 10.1093/cercor/bhz287 31821411

[B14] Dewan M. C. Shults R. Hale A. T. Sukul V. Englot D. J. Konrad P.. (2018). Stereotactic EEG via multiple single-path omnidirectional trajectories within a single platform: Institutional experience with a novel technique. J. Neurosurg. 129, 1173–1181. 10.3171/2017.6.JNS17881 29243976 PMC6003842

[B15] Donos C. Blidarescu B. Pistol C. Oane I. Mindruta I. Barborica A.. (2022). A comparison of uni- and multi-variate methods for identifying brain networks activated by cognitive tasks using intracranial EEG. Front. Neurosci. 16, 946240. 10.3389/fnins.2022.946240 36225734 PMC9549146

[B16] Duñabeitia J. A. Crepaldi D. Meyer A. S. New B. Pliatsikas C. Smolka E.. (2018). MultiPic: a standardized set of 750 drawings with norms for six european languages. Q. J. Exp. Psychol. 71, 808–816. 10.1080/17470218.2017.1310261 28326995

[B17] Ebrahiminia F. Cichy R. M. Khaligh-Razavi S. M. (2022). A multivariate comparison of electroencephalogram and functional magnetic resonance imaging to electrocorticogram using visual object representations in humans. Front. Neurosci. 16, 983602. 10.3389/fnins.2022.983602 36330341 PMC9624066

[B18] Fahrenfort J. J. van Driel J. van Gaal S. Olivers C. N. L. (2018). From ERPs to MVPA using the amsterdam decoding and modeling toolbox (ADAM). Front. Neurosci. 12, 368. 10.3389/fnins.2018.00368 30018529 PMC6038716

[B19] Fischl B. (2012). FreeSurfer. Neuroimage 62, 774–781. 10.1016/j.neuroimage.2012.01.021 22248573 PMC3685476

[B20] Goldman R. N. (1992). Decomposing Linear and Affine Transformations, in Graphics Gems III (IBM Version), ed. Kirk D. B. T. (San Francisco: Morgan Kaufmann), 108–116.

[B21] Gramfort A. Luessi M. Larson E. Engemann D. A. Strohmeier D. Brodbeck C.. (2013). MEG and EEG data analysis with MNE-Python. Front. Neurosci. 7, 267. 10.3389/fnins.2013.00267 24431986 PMC3872725

[B22] Gramfort A. Luessi M. Larson E. Engemann D. A. Strohmeier D. Brodbeck C.. (2014). MNE software for processing MEG and EEG data. Neuroimage 86, 446–460. 10.1016/j.neuroimage.2013.10.027 24161808 PMC3930851

[B23] Grech R. Cassar T. Muscat J. Camilleri K. P. Fabri S. G. Zervakis M.. (2008). Review on solving the inverse problem in EEG source analysis. J. Neuroeng. Rehabil. 5, 25. 10.1186/1743-0003-5-25 18990257 PMC2605581

[B24] Grootswagers T. Wardle S. G. Carlson T. A. (2017). Decoding dynamic brain patterns from evoked responses: a tutorial on multivariate pattern analysis applied to time series neuroimaging data. J. Cogn. Neurosci. 29, 677–697. 10.1162/jocn_a_01068 27779910

[B25] Hatamimajoumerd E. Talebpour A. Mohsenzadeh Y. (2020). Enhancing multivariate pattern analysis for magnetoencephalography through relevant sensor selection. Int. J. Imaging Syst. Technol. 30, 473–494. 10.1002/ima.22398

[B26] Haufe S. DeGuzman P. Henin S. Arcaro M. Honey C. J. Hasson U.. (2018). Elucidating relations between fMRI, ECoG, and EEG through a common natural stimulus. Neuroimage 179, 79–91. 10.1016/j.neuroimage.2018.06.016 29902585 PMC6063527

[B27] Haufe S. Meinecke F. Görgen K. Dähne S. Haynes J. D. Blankertz B.. (2014). On the interpretation of weight vectors of linear models in multivariate neuroimaging. Neuroimage 87, 96–110. 10.1016/j.neuroimage.2013.10.067 24239590

[B28] Haxby J. V. Gobbini M. I. Furey M. L. Ishai A. Schouten J. L. Pietrini P.. (2001). Distributed and overlapping representations of faces and objects in ventral temporal cortex. Science 293, 2425–2430. 10.1126/science.1063736 11577229

[B29] Isnard J. Taussig D. Bartolomei F. Bourdillon P. Catenoix H. Colnat-Coulbois S.. (2018). French guidelines on stereoelectroencephalography (SEEG). Neurophysiol. Clin. 48, 5–13. 10.1016/j.neucli.2017.11.005 29277357

[B30] Jayakar P. Gotman J. Harvey A. S. Palmini A. Tassi L. Schomer D.. (2016). Diagnostic utility of invasive EEG for epilepsy surgery: Indications, modalities, and techniques. Epilepsia 57, 1735–1747. 10.1111/epi.13515 27677490

[B31] Kahane P. Minotti L. Hoffmann D. Lachaux J. P. Ryvlin P. (2003). Invasive EEG in the definition of the seizure onset zone: depth electrodes Handbook of Clinical Neurophysiology (Amsterdam: Elsevier), 109–133. 10.1016/S1567-4231(03)03009-0

[B32] Kappenman E. S. Luck S. J. (2011). The Oxford Handbook of Event-Related Potential Components. Oxford: Oxford University Press

[B33] Koessler L. Cecchin T. Colnat-Coulbois S. Vignal J. P. Jonas J. Vespignani H.. (2015). Catching the invisible: mesial temporal source contribution to simultaneous EEG and SEEG recordings. Brain Topogr. 28, 5–20. 10.1007/s10548-014-0417-z 25432598

[B34] Liang Y. Song C. Liu M. Gong P. Zhou C. Knöpfel T.. (2021). Cortex-wide dynamics of intrinsic electrical activities: propagating waves and their interactions. J. Neurosci. 41, 3665–3678. 10.1523/JNEUROSCI.0623-20.2021 33727333 PMC8055070

[B35] Liu H. Agam Y. Madsen J. R. Kreiman G. (2009). Timing, timing, timing: fast decoding of object information from intracranial field potentials in human visual cortex. Neuron 62, 281–290. 10.1016/j.neuron.2009.02.025 19409272 PMC2921507

[B36] López-Madrona V. J. Medina Villalon S. Badier J. M. Trébuchon A. Jayabal V. Bartolomei F.. (2022). Magnetoencephalography can reveal deep brain network activities linked to memory processes. Hum. Brain Mapp. 43, 4733–4749. 10.1002/hbm.25987 35766240 PMC9491290

[B37] López-Madrona V. J. Pérez-Montoyo E. Álvarez-Salvado E. Moratal D. Herreras O. Pereda E.. (2020). Different theta frameworks coexist in the rat hippocampus and are coordinated during memory-guided and novelty tasks. Elife 9. 10.7554/eLife.57313.sa2 32687054 PMC7413668

[B38] Lubenov E. V. Siapas A. G. (2009). Hippocampal theta oscillations are travelling waves. Nature 459, 534–539. 10.1038/nature08010 19489117

[B39] Mandler G. (1980). Recognizing: the judgment of previous occurrence. Psychol. Rev. 87, 252–271. 10.1037/0033-295X.87.3.252

[B40] Maris E. Oostenveld R. (2007). Nonparametric statistical testing of EEG- and MEG-data. J. Neurosci. Methods 164, 177–190. 10.1016/j.jneumeth.2007.03.024 17517438

[B41] Merkow M. B. Burke J. F. Kahana M. J. (2015). The human hippocampus contributes to both the recollection and familiarity components of recognition memory. Proc. Natl. Acad. Sci. U. S. A. 112, 14378–14383. 10.1073/pnas.1513145112 26578784 PMC4655532

[B42] Mikulan E. Russo S. Parmigiani S. Sarasso S. Zauli F. M. Rubino A.. (2020). Simultaneous human intracerebral stimulation and HD-EEG, ground-truth for source localization methods. Sci. Data 7, 127. 10.1038/s41597-020-0467-x 32345974 PMC7189230

[B43] Mizuseki K. Sirota A. Pastalkova E. Buzsáki G. (2009). Theta oscillations provide temporal windows for local circuit computation in the entorhinal-hippocampal loop. Neuron 64, 267–280. 10.1016/j.neuron.2009.08.037 19874793 PMC2771122

[B44] Muller L. Reynaud A. Chavane F. Destexhe A. (2014). The stimulus-evoked population response in visual cortex of awake monkey is a propagating wave. Nat. Commun. 2014 51 5, 1–14. 10.1038/ncomms4675 24770473 PMC4015334

[B45] Munari C. Hoffmann D. Francione S. Kahane P. Tassi L. Lo Russo G.. (1994). Stereo-electroencephalography methodology: advantages and limits. Acta Neurol. Scand. 152, 56–67. 10.1111/j.1600-0404.1994.tb05188.x 8209659

[B46] Pedregosa F. Varoquaux G. Gramfort A. Michel V. Thirion B. Grisel O.. (2011). Scikit-learn: machine learning in python. J. Mach. Learn. Res. 12, 2825–2830.

[B47] Pistol C. Daneasa A. Ciurea J. Rasina A. Barborica A. Oane I.. (2021). Accuracy and safety of customized stereotactic fixtures for stereoelectroencephalography in pediatric patients. Stereotact. Funct. Neurosurg. 99, 17–24. 10.1159/000510063 33227801

[B48] Pizzo F. Roehri N. Medina Villalon S. Trébuchon A. Chen S. Lagarde S.. (2019). Deep brain activities can be detected with magnetoencephalography. Nat. Commun. 10, 971. 10.1038/s41467-019-08665-5 30814498 PMC6393515

[B49] Postelnicu G. Zollei L. Fischl B. (2009). Combined volumetric and surface registration. IEEE Trans. Med. Imaging 28, 508–522. 10.1109/TMI.2008.2004426 19273000 PMC2761957

[B50] Ramon C. Schimpf P. H. Haueisen J. (2006). Influence of head models on EEG simulations and inverse source localizations. Biomed. Eng. Online 5, 10. 10.1186/1475-925X-5-10 16466570 PMC1389789

[B51] Ratcliff R. Sederberg P. B. Smith T. A. Childers R. (2016). A single trial analysis of EEG in recognition memory: tracking the neural correlates of memory strength. Neuropsychologia 93, 128–141. 10.1016/j.neuropsychologia.2016.09.026 27693702 PMC5148728

[B52] Ray A. Tao J. X. Hawes-Ebersole S. M. Ebersole J. S. (2007). Localizing value of scalp EEG spikes: a simultaneous scalp and intracranial study. Clin. Neurophysiol. Off. J. Int. Fed. Clin. Neurophysiol. 118, 69–79. 10.1016/j.clinph.2006.09.010 17126071

[B53] Rutishauser U. Mamelak A. N. Schuman E. M. (2006). Single-trial learning of novel stimuli by individual neurons of the human hippocampus-amygdala complex. Neuron 49, 805–813. 10.1016/j.neuron.2006.02.015 16543129

[B54] Sassenhagen J. Draschkow D. (2019). Cluster-based permutation tests of MEG/EEG data do not establish significance of effect latency or location. Psychophysiology 56, e13335. 10.1111/psyp.13335 30657176

[B55] Schoffelen J. M. Gross J. (2009). Source connectivity analysis with MEG and EEG. Hum. Brain Mapp. 30, 1857–1865. 10.1002/hbm.20745 19235884 PMC6870611

[B56] Steinmetz N. A. Aydin C. Lebedeva A. Okun M. Pachitariu M. Bauza M.. (2021). Neuropixels 2, 0. A miniaturized high-density probe for stable, long-term brain recordings. Science 372, eabf4588. 10.1126/science.abf4588 33859006 PMC8244810

[B57] Tang A. C. Liu J. Y. Sutherland M. T. (2005). Recovery of correlated neuronal sources from EEG: the good and bad ways of using SOBI. Neuroimage 28, 507–519. 10.1016/j.neuroimage.2005.06.062 16139528

[B58] Tao J. X. Ray A. Hawes-Ebersole S. Ebersole J. S. (2005). Intracranial EEG substrates of scalp EEG interictal spikes. Epilepsia 46, 669–676. 10.1111/j.1528-1167.2005.11404.x 15857432

[B59] Van Veen B. D. van Drongelen W. Yuchtman M. Suzuki A. (1997). Localization of brain electrical activity via linearly constrained minimum variance spatial filtering. IEEE Trans. Biomed. Eng. 44, 867–880. 10.1109/10.623056 9282479

[B60] Wager T. D. Kang J. Johnson T. D. Nichols T. E. Satpute A. B. Barrett L. F.. (2015). A bayesian model of category-specific emotional brain responses. PLOS Comput. Biol. 11, e1004066. 10.1371/journal.pcbi.1004066 25853490 PMC4390279

[B61] Wardle S. G. Kriegeskorte N. Grootswagers T. Khaligh-Razavi S. M. Carlson T. A. (2016). Perceptual similarity of visual patterns predicts dynamic neural activation patterns measured with MEG. Neuroimage 132, 59–70. 10.1016/j.neuroimage.2016.02.019 26899210

[B62] Westner B. U. Dalal S. S. Gramfort A. Litvak V. Mosher J. C. Oostenveld R.. (2022). A unified view on beamformers for M/EEG source reconstruction. Neuroimage 246, 118789. 10.1016/j.neuroimage.2021.118789 34890794

[B63] Yonelinas A. P. (2002). The nature of recollection and familiarity: a review of 30 years of research. J. Mem. Lang. 46, 441–517. 10.1006/jmla.2002.286416899208

[B64] Yu H. Pistol C. Franklin R. Barborica A. (2018). Clinical accuracy of customized stereotactic fixtures for stereoelectroencephalography. World Neurosurg. 109, 82–88. 10.1016/j.wneu.2017.09.089 28951181

