# Supplementary material for: Studying memory processes at different levels with simultaneous depth and surface EEG recordings
Source: Front Hum Neurosci. 2023 Apr 4;17:1154038. doi: 10.3389/fnhum.2023.1154038 (PMC10110965; doi:10.3389/fnhum.2023.1154038)
Supplement: Supplementary file 1 [file Data_Sheet_1.PDF]

Supplementary Material

Studying memory processes at different levels with simultaneous depth and surface EEG recordings

Andrei Barborica*, Ioana Mindruta, Víctor J López-Madrona, F.-Xavier Alario, Agnès Trébuchon, Cristian Donos, Irina Oane, Constantin Pistol, Felicia Mihai, Christian G. Bénar *

*** Correspondence:**
Andrei Barborica
andrei.barborica@fizica.unibuc.ro

Christian G. Bénar
christian.benar@univ-amu.fr

Supplementary Table S1. Abbreviations used for manual labeling of the intracranial electrode contacts.

Area	Abbreviation	Location
MESIAL TEMPORAL	A	Amygdala
	Hc	Hippocampus
LATERAL TEMPORAL	TP	Temporal Pole
	STG	Superior Temporal Gyrus
	MTG	Middle Temporal Gyrus
	ITG	Inferior Temporal Gyrus
	W	Wernicke
INFERIOR TEMPORAL	F	Fusiform Gyrus
	PHG	Parahippocampal Gyrus
	LG	Lyngual Gyrus
	E	Entorhinal
LATERAL PARIETAL	SPL	Superior Parietal Lobule
	IPL	Inferior Parietal Lobule
	S	Postcentral Gyrus
	AG	Angular Gyrus
	SMG	Supramarginal Gyrus
	IPS	Intraparietal sulcus
MEDIAL PARIETAL	PrC	Pre-Cuneus
	PCL	Paracentral Lobule
	PCC	Posterior Cingulate
	Ist	Isthmus
OCCIPITAL	O	Lateral Occipital

	TPO	Temporo-Parieto Occipital
	V1	Primary Visual Cortex
	C	Cuneus
**INSULA**	aI	Anterior Insula
	pI	Posterior Insula
	OpF	Operculum Frontalis
	OpR	Operculum Rolandis
	OpP	Operculum Parietalis
	OpT	Operculum Temporalis
**LATERAL FRONTAL**	R	Rolandic
	B	Broca
	PMC	PreMotor
	DLPFC	Dorso-Lateral Prefrontal
	VLPFC	Ventro-Lateral Prefrontal
	OF	Orbitofrontal
**MESIAL FRONTAL**	SMA	Supplementary Motor Area
	preSMA	Pre-Supplementary Motor Area
	SFG	Superior Frontal Gyrus
	MOFC	Medial Orbito-Frontalis
	FP	Frontal Pole
	ACC	Anterior Cingulate
	MCC	Middle Cingulate
	DMPFC	Dorso-Medial Prefrontal Cortex
	VMPFC	Ventro-Medial Prefrontal Cortex
**OTHER**	WM	White matter
	BG	Basal ganglia
